# BSA-Seq and Fine Linkage Mapping for the Identification of a Novel Locus (*qPH9*) for Mature Plant Height in Rice (*Oryza sativa*)

**DOI:** 10.1186/s12284-022-00576-2

**Published:** 2022-05-20

**Authors:** Wei Xin, HuaLong Liu, Luomiao Yang, Tianze Ma, Jingguo Wang, Hongliang Zheng, Wenxing Liu, Detang Zou

**Affiliations:** Key Laboratory of Germplasm Enhancement, Physiology and Ecology of Food Crops in Cold Region, Ministry of Education, Northeast Agricultural University, Harbin, 150030 China

**Keywords:** *Oryza sativa* L., Plant height, BSA-seq, Linkage-mapping, Quantitative trait locus

## Abstract

**Background:**

Plant height is a key factor in the determination of rice yield since excessive height can easily cause lodging and reduce yield. Therefore, the identification and analysis of plant height-related genes to elucidate their physiological, biochemical, and molecular mechanisms have significant implications for rice breeding and production.

**Results:**

High-throughput quantitative trait locus (QTL) sequencing analysis of a 638-individual F2:3 mapping population resulted in the identification of a novel height-related QTL (*qPH9*), which was mapped to a 2.02-Mb region of Chromosome 9. Local QTL mapping, which was conducted using 13 single nucleotide polymorphism (SNP)-based Kompetitive allele-specific PCR (KASP) markers for the *qPH9* region, and traditional linkage analysis, facilitated the localization of *qPH9* to a 126-kb region that contained 15 genes. Subsequent haplotype and sequence analyses indicated that *OsPH9* was the most probable candidate gene for plant height at this locus, and functional analysis of *osph9* CRISPR/Cas9-generated *OsPH9* knockout mutants supported this conclusion.

**Conclusion:**

*OsPH9* was identified as a novel regulatory gene associated with plant height in rice, along with a height-reducing allele in ‘Dongfu-114’ rice, thereby representing an important molecular target for rice improvement. The findings of the present study are expected to spur the investigation of genetic mechanisms underlying rice plant height and further the improvement of rice plant height through marker-assisted selection.

**Supplementary Information:**

The online version contains supplementary material available at 10.1186/s12284-022-00576-2.

## Introduction

Plant height is a key factor in determining rice yield since excessive height can easily cause lodging and reduce yield. As such, the breeding and large-scale promotion of semi-dwarf rice varieties, which has been conducted since the 1950s, has increased rice yield by 20–30% and has, accordingly, been hailed as the "green revolution" in rice production. The first major breakthrough was attributed to the successful application of a semi-dwarf gene (Peng et al. 1999; Hedden 2003).In the 1970s and 1980s, three-line rice breeding aimed at promoting heterosis resulting in another leap in rice yield and progression towards ensuring China’s food security. Nowadays, semi-dwarfing materials are also used in the successful development of hybrid rice. However, in the 30 years succeeding the two major leaps in dwarf and hybrid rice breeding, the yield of rice in China has remained more or less stagnant.

Super rice breeding with ideal plant type as the model represents an important strategy for promoting future improvements in China’s rice yield. The main strategy of such breeding efforts is to combine ideal plant type with heterosis (Chen et al. 2001). Plant type improvement is the main aim of rice breeding and is highly dependent on plant height. However, the semi-dwarf varieties that are currently used in production are all associated with the recessive dwarf gene *Ossd1*, and the wide application of this single gene poses a potential risk of genetic diversity loss. At the same time, rice varieties that carry the semi-dwarf gene *sd1* also exhibit poor drought tolerance and low photosynthetic effect, which has become a bottleneck in the development of new rice varieties (Sha et al. 2021). Therefore, the identification and analysis of plant height-related genes, to elucidate their physiological, biochemical, and molecular mechanisms, have significant implications for rice breeding and production.

Rice plant height is a quantitative trait controlled by multiple genes, that are associated with a variety of physiological traits and processes. In fact, at least 70 dwarf mutants of rice have been discovered, and the underlying mechanisms have reportedly been associated with the phytohormone signaling and the biosynthesis of gibberellic acid (GA), abscisic acid (ABA), and various brassinosteroids (Spielmeyer et al. 2002; Sakamoto et al. 2004; Hubbard et al. 2010; Sun 2010; Song et al. 2012; Shen et al. 2014; Tong et al. 2014; Liu et al. 2015; Wu et al. 2020; Lin et al. 2020); as well as transcriptional regulation, which plays an important regulatory role in rice growth and development (Tan et al. 2008; Yaish et al. 2010; Wu et al. 2020; Wei et al. 2021). The gene *IPA1*, for example, which encodes a transcription factor that contains the SBP-box domain, has been reported to regulate multiple growth and developmental processes, including the formation of an ideal plant type, and loss-of-function mutants characterized by desirable agronomic traits; such as fewer ineffective tillers, strong stalks, lodging resistance, large panicles with more grains, and high yield (Zhang et al. 2017; Liu et al. 2019; Wang et al. 2020). Meanwhile, Wei et al. (2021) reported the localization and cloning of the plant height-related WRKY-family transcription factor *WRKY21*. Thus, mining for novel plant height genes is likely an effective strategy for further constructing an ideal plant type and increasing yield.

One effective method for the identification of novel height-related genes is the use ofQTL linkage analysis. Traditional QTL mapping usually involves the genotyping of numerous individuals from a population using molecular markers distributed across the whole genome (Zou et al. 2016). However, to ensure sufficient statistical power, this strategy requires the genotype and phenotype analysis of numerous offsprings, which is both time-consuming and labor-intensive. In contrast, bulked segregant analysis (BSA) only requires the genotyping of individuals with extreme phenotypes (Giovannoni et al. 1991; Michelmore et al. 1991). Initially, BSA was widely used in QTL identification and gene mining related to specific traits; such as disease resistance, color, and fertility (Zhang et al. 1994, 1996; Monna et al. 1995). However, the recent and rapid development of next-generation sequencing (NGS) technology has enabled the use of BSA, along with whole-genome sequencing (i.e., BSA-seq), to efficiently identify QTLs and trait-related genes (Abe et al. 2012; Kadambari et al. 2018). In comparison to traditional QTL mapping, BSA-seq ensures improved work efficiency and sufficient statistical power and has been successfully applied to a variety of plant taxa; including arabidopsis, rice, maize, and pepper (Ramirez-Gonzalez et al. 2015; Huang et al. 2017; Zegeye et al. 2018). However, because the method yields less-than-ideal confidence interval resolution, researchers have had to combine BSA-seq with fine mapping to identify potential candidate genes for specific QTLs (Wambugu et al. 2018). For instance, the QTL qRSL7 (Lei et al. 2020) was mapped from 4.17 Mb to 222 kb by BSA-seq and classical QTL mapping to allow the identification of final candidate genes. In addition, the gene *ZmVEN1*, which is associated with maize grain texture, was detected using BSA-seq and RNA-seq (Wen et al. 2019). Thus, the integration of BSA-seq and fine mapping are necessary for locating major QTLs and mining target genes.

Accordingly, the aim of the present study was to implement BSA-seq and fine mapping for the identification of height-related genes in rice. An F2:3 population derived from a cross of tall and dwarf varieties (Longyang11 and Dongfu114, respectively) was subjected to BSA-seq analysis and fine-mapping strategy, along with haplotype and sequence analysis, to identify potential candidate genes. Further, CRISPR/Cas9 gene editing was used to develop a knockout mutant to assess the function of the putative height-related genes.

## Materials and Methods

### Plant Materials and Height Evaluation

The japonica varieties ‘Dongfu 114’ (‘DF114’) and ‘Longyang 11’ (‘LY11’) were obtained from Northeast Agriculture University (Harbin, China) and used as female and male parents, respectively, to generate an F_2:3_ population of 638 individuals. In the spring of 2019, ‘DF114’ (n = 48), ‘LY11’ (n = 48), and F_2:3_ (n = 638) individuals were planted in four rows under natural conditions in paddy fields at Acheng Experimental Station (Heilongjiang Province, China), and 5 plants from the center of each plot were selected for plant height evaluation.

### Construction of Segregating Pools

All flag leaves of the 638 F_2:3_ individuals were collected separately for total genomic DNA extraction, which was performed using the Cetyltrimethylammonium bromide (CTAB) method (Murray et al. 1980), with minor modifications, and the isolated DNA was quantified using a Nanodrop 2000 spectrophotometer (Thermo Fisher Scientific, Waltham, MA, USA). After quantification, a Qubit 2.0 fluorimeter (Life Technologies, Carlsbad, CA, USA) was used to pool equimolar amounts of genomic DNA from the 30 shortest and 30 tallest individuals (L-pool and H-pool, respectively).

### Whole-Genome Re-Sequencing

Total genomic DNA was extracted from the bulked pools, and at least 3 µg of genomic DNA was used to construct paired-end libraries, with an insert size of 500 bp, using the Illumina paired-end DNA sample prep kit (San Diego, CA, USA). The resulting libraries were sequenced by Genedenovo (Guangzhou, China) using the HiSeq X10 NGS platform (Illumina). To achieve quality trimming, thereby ensuring high-confidence variant calling, raw reads were filtered by removing reads with ≥ 10% unidentified nucleotides, with > 50% bases with shared quality scores of ≤ 20, or aligned to the barcode adapter. To identify SNPs and indels, filtered reads were aligned to the ‘Nipponbare’ reference genome sequence (Matsumoto et al. 2005) using the Burrows-Wheeler Aligner (v.0.7.16a-r1181; Kumar et al. 2019), using *‘mem -M’* setting, where *-M* is an option used to mark shorter split-alignment hits as secondary alignments. Variant calling was performed using the GATK Unified Genotyper (v.3.5; https://gatk.broadinstitute.org), and both SNPs and indels were filtered using the GATK Variant Filtration function with proper standards (-Window 4, -filter "QD < 4.0 || FS > 60.0 || MQ < 40.0 ", -G_filter "GQ < 20"). All mutations for genes, functions, and genomic regions were annotated using ANNOVAR (Wang et al. 2010). Association analysis was performed using, ∆(SNP-Index) (Abe et al. 2012; Takagi et al. 2013), G-value (Magwene et al. 2011; Mansfeld et al. 2018), ED (Hill et al. 2013), and two-tailed Fisher’s exact test (Fisher et al. 1922) values; the overlapping interval of the four methods was considered as the final QTL interval.

### Development of SNP Markers and Narrowing Candidate Interval

KASP markers were designed using Premier 5.0 **(**Additional file 1: Table S4), and markers that were polymorphic between parents were used to validate BSA-seq results and construct a linkage map, by genotyping the 638 progeny individuals, and to narrow the potential candidates using the Inclusive Composite Interval Mapping (ICIM) module of QTL IciMapping (v.4.2; http://www.isbreeding.net). The Logarithm of the odds (LOD) score threshold for confirming a significant QTL was established using a permutation test with 1000 replicates and a significant level of *P* < 0.01.

### Knockout Plant Construction

CRISPR/Cas9 gene-editing vector construction was conducted as described by Li et al. (2017). Two target sequences, including Point accepted mutation (PAM) (GGCAAGGGAGGGAAGGGTCTCGG / CGTCTACGCCCTCAAGCGCCAGG) were selected within the target genes, and the targeting specificity was confirmed using a BLAST search against the rice genome (Hsu et al. 2013). Genomic DNA was extracted from these knockout lines; and after PCR amplification, the designed target site amplicon (300–500 bp) was sequenced directly and identified using the Degenerate Sequence Decoding method (Ma et al. 2015). Knockout lines were confirmed by PCR sequencing, with the primers 5’-CCCAATACAGATCAACCAAA-3’ and 5’-AGAACTGAACTACAGCAAGT-3’. The knockout lines were potted (inner diameter 30 cm), along with the control (WT) variety ‘Dongnong 430’, in the spring of 2021 at Northeast Agricultural University (Harbin, China), and upon reaching maturity, the height of each line (n = 5) was evaluated.

### Statistical Analysis

Differences between parent and progeny plant height were detected using SPSS18.0 (IBM, Armonk, NY, USA). Data represent means ± standard deviations. Graphs were drawn using edgeR (http://www.r-project.org/) and Origin 2018 (OriginLab, Northampton, MA, USA).

## Results

### Screening and Evaluation of Plant Height

**Fig. 1:**
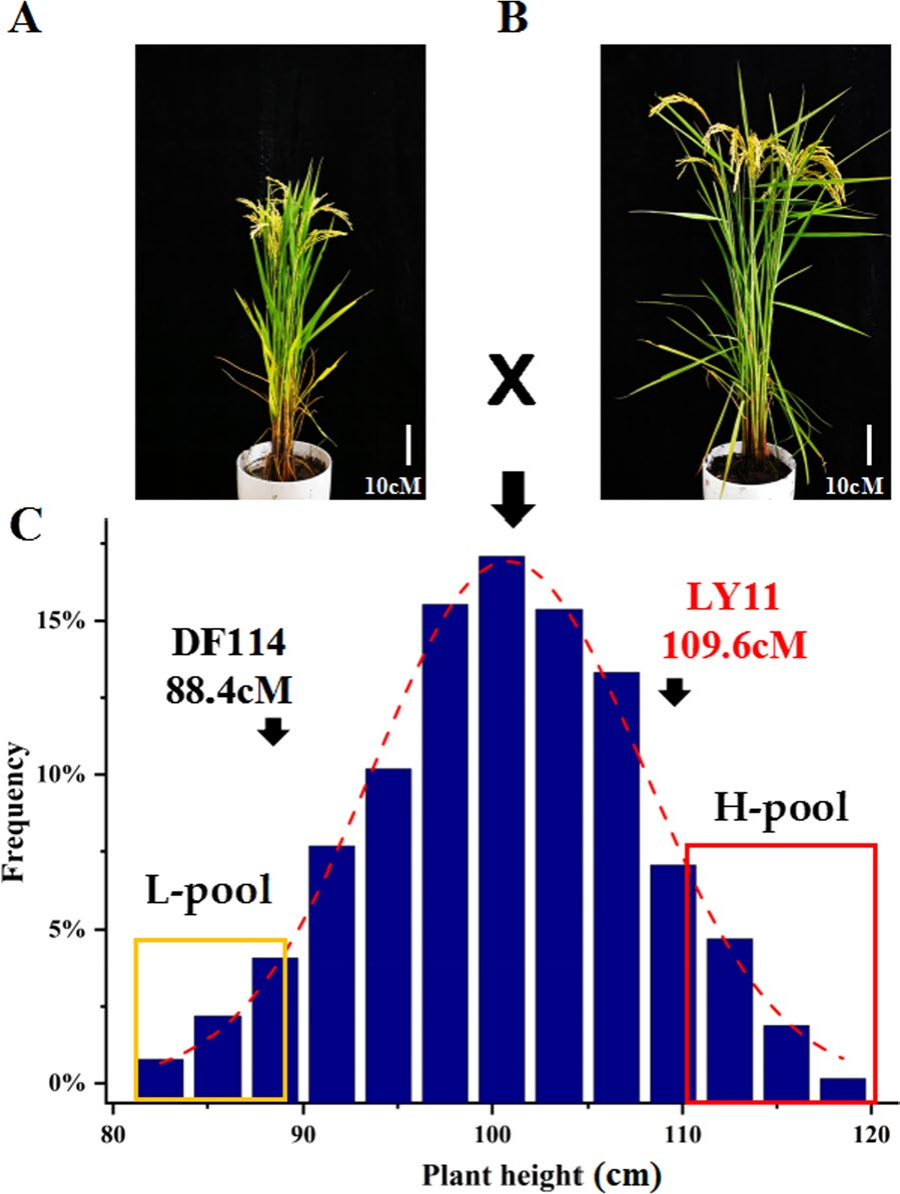
Phenotype analysis of plant height in the progeny of a cross between ‘DF114’ and ‘LY11’. **A** Mature ‘DF114’ plant. **B** Mature ‘LY11’ plant. **C** Distribution of plant height among 638 mapping individuals of F2:3 population

Of the two parental varieties, ‘DF114’ plants were shorter than ‘LY11’ plants **(**Fig. 1A, B), and the heights of the 628 F_2:3_ progenies (Fig. 1C) ranged from 82.5 to 117.3 cm, with the 30 shortest and tallest progenies assigned to the L-pool and H-pool, respectively, for DNA resequencing. In addition, both the skewness and kurtosis associated with plant height in the F_2:3_ population were close to 1 (Additional file 1: Table S1), which indicated that the data were suitable for QTL analysis.

### Whole-Genome Resequencing and BSA-Seq Analysis

**Fig. 2:**
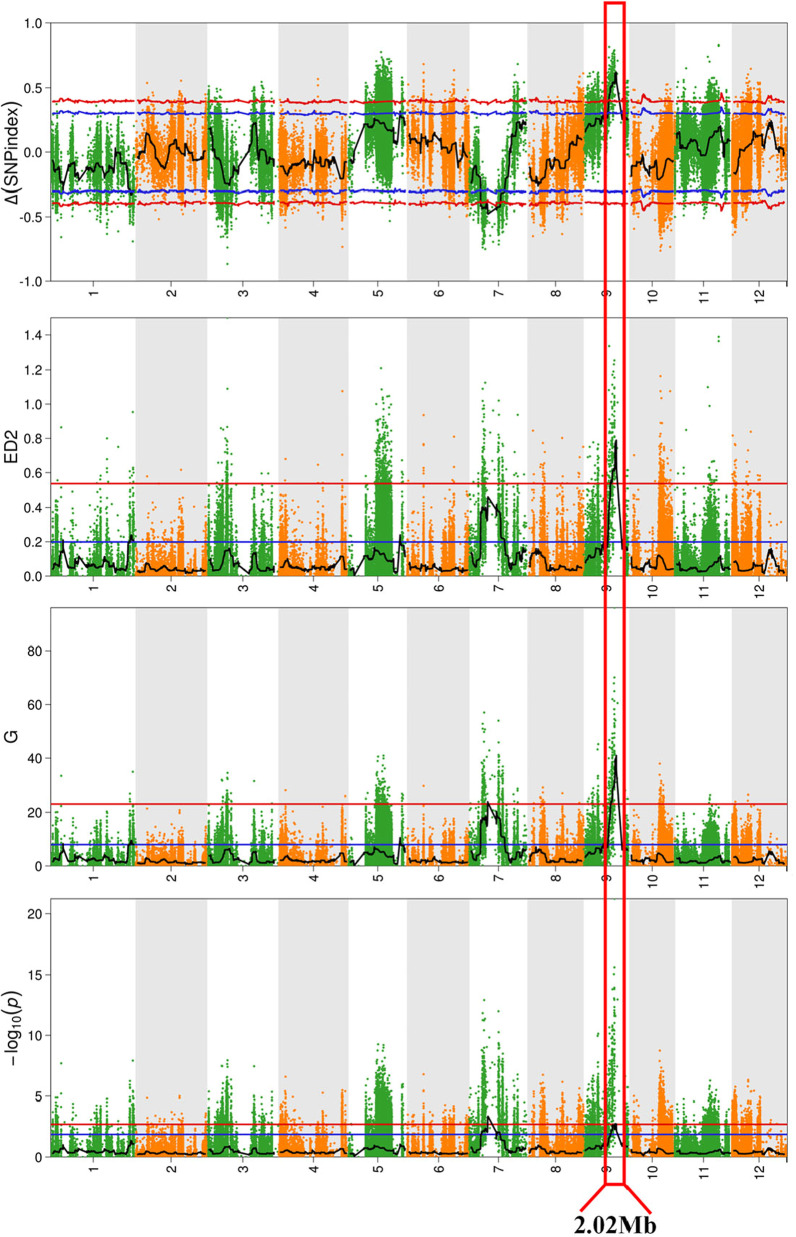
Quantitative trait locus (QTL) analysis of rice plant height at maturity using 4 QTL-seq methods. **A** Manhattan plot showing the distribution of Δ(SNP-index) on chromosomes. **B** Manhattan plot showing the distribution of Euclidean distance (ED2) on chromosomes. **C** Manhattan plot showing the distribution of G-value on chromosomes. **D** Manhattan plot showing the distribution of log-transformed Fisher’s exact test P-value distribution, –log10(p) on chromosomes. Blue and red lines represent 95 and 99% confidence intervals, respectively, and black lines represent mean values of the 4 algorithms, which were drawn using sliding window analysis. Numbers on the horizontal coordinates represent chromosome numbers

The mean coverage depth for the parents and the two pools was 50 × , and comparison of the sequences to the ‘Nipponbare’ reference genome resulted in the identification of 801,247 SNPs and 133,510 indels, which were reduced to 277,729 SNPs and 45,142 indels by trimming and filtering (Additional file 1: Table S2). A total of 322,871 high-quality SNPs/indels that were homozygous in each parent and polymorphic between the parents were then selected for BSA-seq analysis, using ∆(SNP-Index) values **(**Fig. 2A), Euclidean distance (ED) values (Fig. 2B), and G-values (Fig. 2C), as well as Fisher’s exact test *P*-values (Fig. 2D), were used to identify candidate plant height-related QTL regions (Table 1). Two significant (*P* < 0.01) peaks in the ∆(SNP-Index) distribution spanned 12.14-Mb (4.78–16.92 Mb) and 5.98-Mb (11.82–17.80 Mb) intervals (*qPH7* and *qPH9*) on chromosomes 7 and 9, respectively, and significant peaks in G-value distribution completely covered the results of ∆(SNP-Index) analysis, whereas those in the ED and Fisher’s exact test *P*-value distributions completely covered the interval on Chromosome 9 alone. Furthermore, only the region identified in the Fisher’s exact test *P*-value distribution (2.02 Mb of *qPH9*) was included in the intersection of genome regions identified by all four methods. It is also worth noting that, in the Δ(SNP-Index) and ED value distributions, the peak values observed for *qPH9* were higher than those observed for *qPH7* **(**Table 1), which indicated a greater difference in the allele ratio between the two mixed pools. Therefore, *qPH9* was considered to be a more significant target for mining candidate plant height genes. There is a known panicle gene *DEP1* in the *qPH9* interval, and the *DEP1* genome sequence analysis shows that there is no difference between ‘DN114’ and ‘LY11’ (Additional file 2: Fig. S1).

**Table 1:** QTLs conferring plant height at maturity by 4 methods for identification using BSA-seq

Method	QTL name	Chr	Start (bp)	End (bp)	Peak	Overlapping region (Mb)
Δ(SNP-index)	qPH7	7	4,780,001	16,920,000	−0.4781	4.78–16.92
	qPH9	9	11,820,001	17,800,000	0.6201	11.82–17.80
ED-Value	qPH9	9	12,740,001	17,800,000	0.7888	12.74–17.80
G-Value	qPH7	7	8,380,001	10,520,000	23.9234	8.38–10.52
	qPH9	9	12,800,001	17,800,000	40.9305	12.80–17.80
Fish-*P*-Value	qPH9	9	15,660,001	17,680,000	0.0090	15.66–17.68

*Chr* chromosome, *ED* Euclidian distance, *Fish-P-value* Fisher’s exact test *P*-value

### Fine Mapping of *qPH9*

**Fig. 3:**
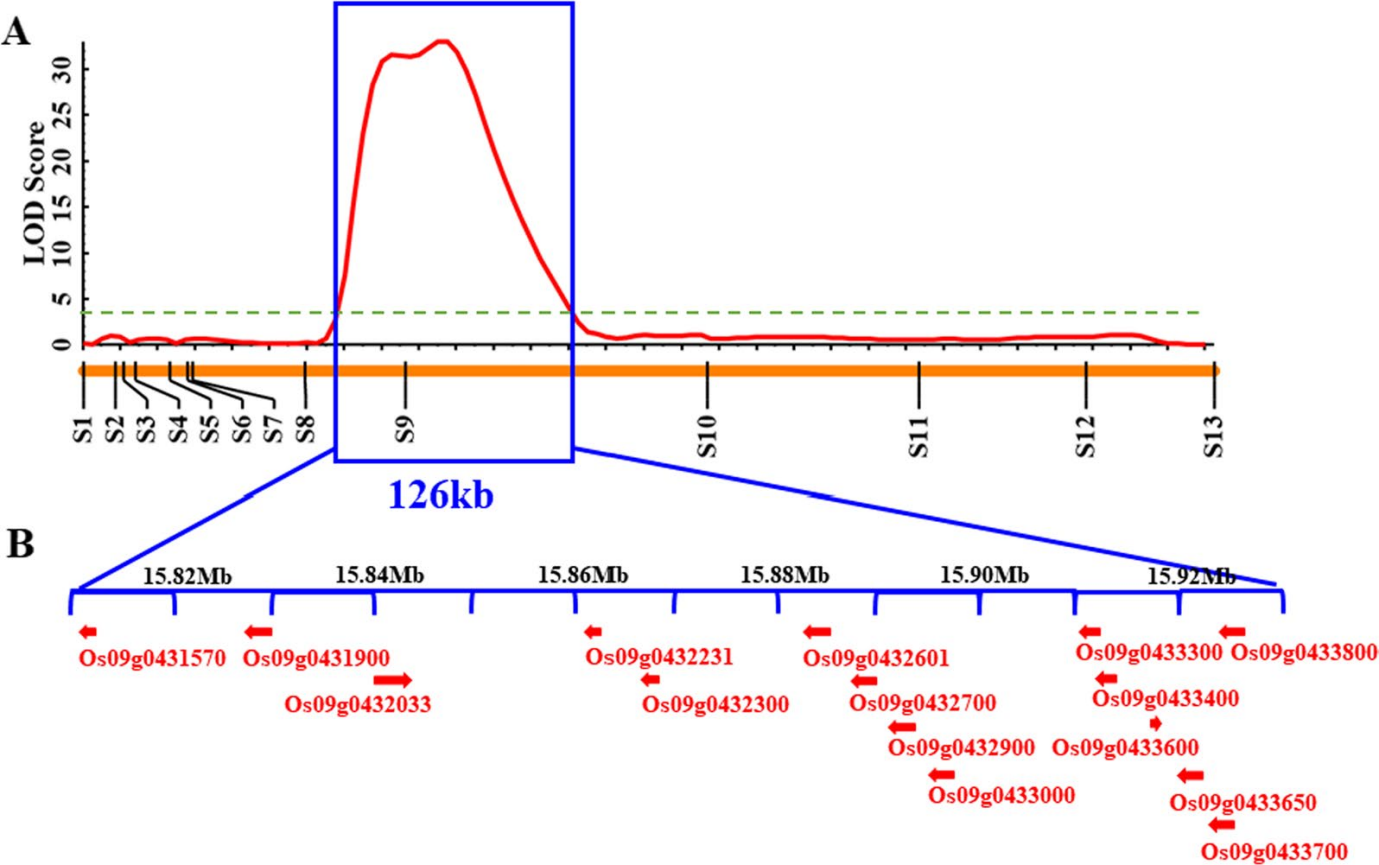
Fine mapping of the quantitative trait locus (*qPH9*) associated with rice plant height at maturity. **A** Detection of *qPH9* by ICIM module of QTL IciMapping 4.2. The orange bar with vertical black lines and labels represents the linkage map and Kompetitive allele-specific PCR (KASP) markers. **B** Putative plant height genes identified at *qPH9* using annotation information from the ‘Nipponbare’ reference genome (http://plants.ensembl.org/index.html/)

The *qPH9* coding region harbored 26 SNPs and 14 indels (Additional file 1: Table S3). The analysis of 13 highly credible SNPs (Additional file 1: Table S4) in the 638 progenies, using a LOD score of 3.0 as a threshold for consecutive occurrence, yielded a *qPH9* linkage map (Fig. 3A). The *qPH9* locus was simultaneously linked to plant height and anchored to the 126 kb interval between 15,803,211 bp and 15,929,211 bp (Fig. 3B, Additional file 1: Table S5), which explained 20.50% of the phenotypic variation in plant height, and the peak LOD score was 32.95 (Table 2). The positive *qPH9* allele was contributed by the ‘DF114’parent.

**Table 2:** Identification of *qPH9* for plant height at maturity by linkage analysis

Trait	QTL	Chromosome	Position	Left Marker	Right Marker	LOD	PVE (%)	Add
PH	*qPH9*	9	15,862,211	S8	S10	32.95	20.50	− 7.81

*Add* additive effects, *LOD* logarithm of the odds, *PVE* phenotypic variation explained, *QTL* quantitative trait locus

### Putative Candidate Genes for *qPH9*

Annotation of the 126 kb interval, using annotation information of the ‘Nipponbare’ reference genome (http://plants.ensembl.org/index.html/), resulted in the identification of 15 candidate genes (Fig. 3B). These genes include 4 hypothetical proteins, 2 non-coding proteins, 2 C2H2 transcription factor family proteins, 2 Glycosyl transferase proteins, 1 Cold-regulated protein, 1 AAA-type ATPase family protein, 1 Histone H4 protein, 1 Pectin methylesterase and 1 Senescence-associated protein (Additional file 1: Table S6). The resequencing data (Additional file 1: Table S3) indicated that the difference between ‘DN114’ and ‘LY11’ were in the 15,803,211 bp and 15,929,211 position of Chromosome 9, which was within the range of 126 kb. Among them, SNP (9:15,916,244) is a non-synonymous mutation in the CDs region of *Os09g0433600*. In addition, the genome sequence of *Os09g0433600* also showed that, there were only SNP differences at 9:15,916,244 between *Os09g0433600* of ‘DN114’ and ‘LY11’ (Additional file 3: Fig. S2). Therefore, we believe that *Os09g0433600* is a candidate gene for *qPH9*, and named *OsPH9.*

### Significant Association SNP of *OsPH9* with Plant Height by RFGB Database

**Fig. 4:**
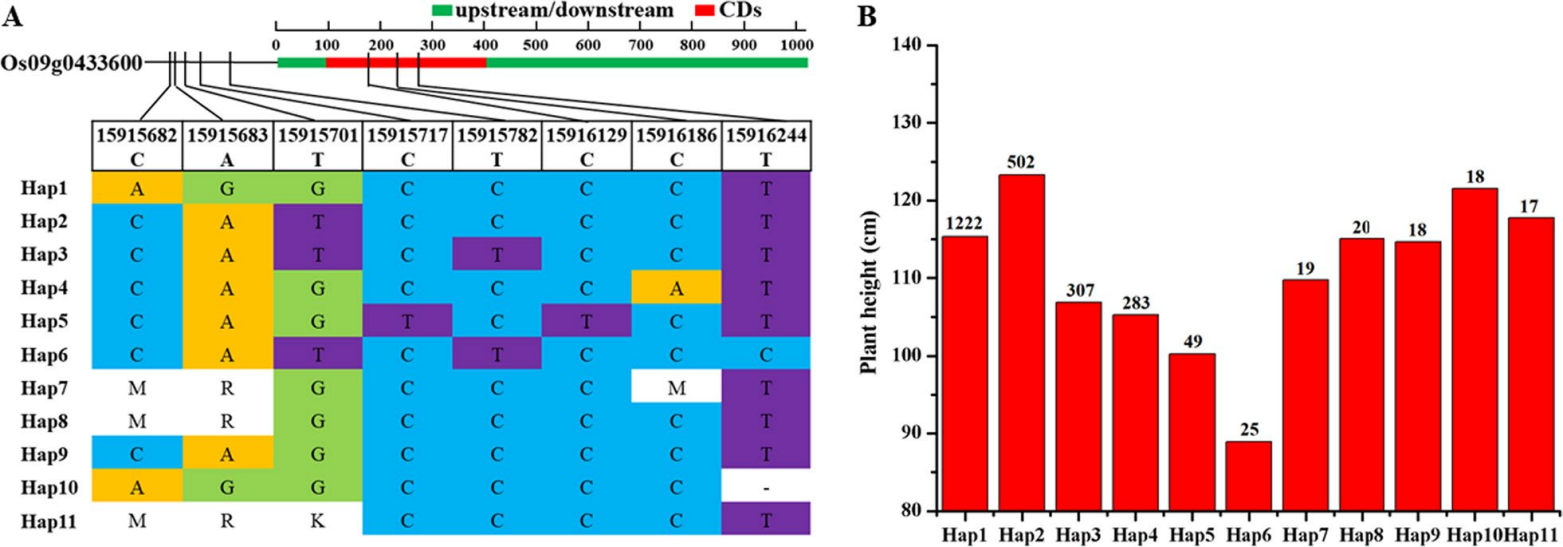
Haplotype and sequence analysis. **A** Haplotype analysis of Os09g0433600, **B** Plant height differences of different haplotypes

With RFGB Database analysis **(**Fig. 4; Additional file 1: Table S7), 8 SNPs were obtained in the promoter region and CDs region of *OsPH9*, which were 15,915,682 (C > A/M), 15,915,683 (A > G/R), 15,915,701 (T > G/K), 15,915,717C (C > T) and 15,915,782 (T > C) in the promoter region, and 15,916,129 (C > T), 15,916,186 (C > A/M) and 15,916,244 (T > C/-) in the CDs region, respectively. A total of 11 haplotypes were obtained based on the different combinations of 8 SNPs. As shown in Fig. 4B, the plant height difference between haplotypes is significant, which indicates that the sequence difference of *OsPH9* is closely related to the change in plant height. Among them, 15,916,244 (T > C) is consistent with resequencing results (Additional file 1: Table S3), indicating that 15,916,244 (T > C) may be the main reason for the difference in plant height between ‘DF114’ and ‘LY11’. We then referred to the data from the 3010 Rice Genomes Project and found that 15,916,244C mainly exists in the genotype of japonica, its allele frequency was 2.82% in japonica; however, it did not occur in indica. It could thus be inferred that 15,916,244C is a rare natural variation (Additional file 1: Table S8). Therefore, it can be used as a special functional variation in ‘DF114’ to improve the plant height of rice varieties.

### Knockout of *OsPH9* using CRISPR/Cas9 System

**Fig. 5:**
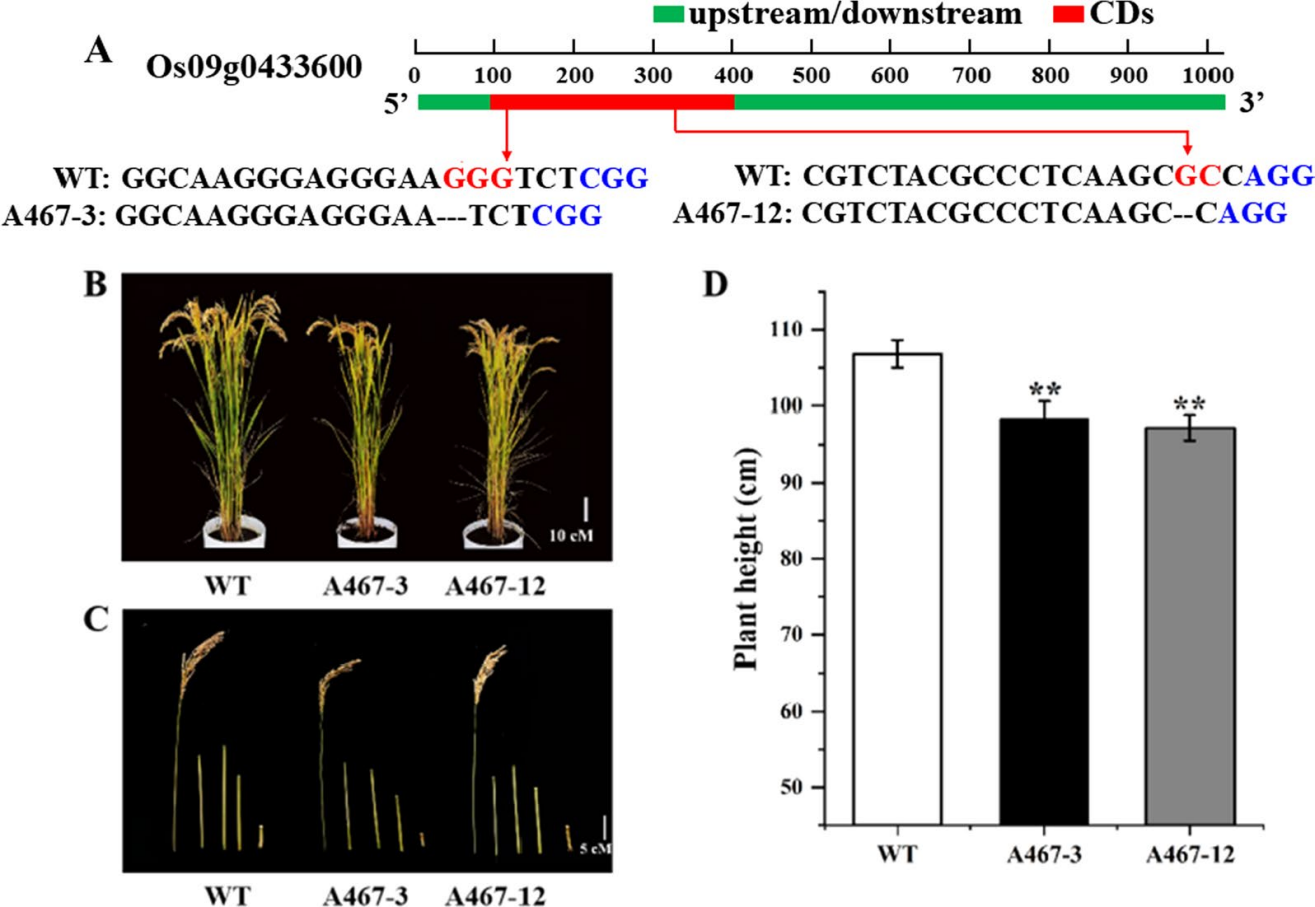
Functional analysis of the H4 histone coding gene Os09g0433600 (OsPH9) in rice. **A** DNA sequences of Os09g0433600 in ‘Dongnong 430’ (WT) and knockout lines (A467-3 and A467-12). **B** Mature plant height phenotypes of WT and knockout mutant lines. **C** Stem node lengths of WT and knockout mutant lines. **D** Mature plant heights of WT and knockout mutant lines (**P < 0.01; Student’s *t*-test)

Functional analysis of the H4 histone coding gene *Os09g0433600* (*OsPH9*), was conducted using the CRISPR/Cas9-generated *osph9*-mutant lines (A467-3 and A467-12) in which the motifs GGG and GC were deleted at the 26 and 247 bp of the CDs region, respectively (Fig. 5A). This revealed that the loss-of-function of *OsZOS9-12* resulted in reduced height and node length (Fig. 5B,C), with the mature plant height of lines A467-3 and A467-12 being reduced by 7.96 and 8.99%, respectively, when compared to the wild-type plants (Fig. 5D).

## Discussion

Plant height has been a major breeding target in rice, due to the importance of lodging resistance and mechanized harvesting requirements. Many dwarf and semi-dwarf genes have been identified in rice. For example, mutations in *OsBR6ox*, which encodes a key enzyme in brassinosteroid biosynthesis, and in genes that regulate GA synthesis (e.g., *SD1*, *D18*, *D35*) leading to varying degrees of dwarfing (Hong et al. 2002; Monna et al. 2002; Tong et al. 2014; Wu et al. 2014). However, the continuous mining of novel height-related genes is essential for developing an ideal rice plant type and for improving rice yield. Thus, the aim of the present study was to investigate the genetic basis for short plant height in ‘DF114’ rice, thereby establishing a theoretical foundation for future plant type improvement via integrated BSA-seq and linkage mapping analysis.

Along with the recent development and application of high-throughput sequencing, high-resolution mass spectrometry, and information processing, BSA-seq has gradually matured. Its accuracy and cost have improved significantly. The combination of traditional mapping and BSA-seq can effectively and quickly reduce the major QTL interval (Takagi et al. 2015L, Lei et al. 2020; Yang et al. 2021). For instance, Zhang et al. (2021) used BSA-seq to identify a major QTL (*qPH8*) in a large rice population with an effect equivalent to that of *SD1*. In the present study, BSA-seq was used to identify a major QTL (*qPH9*), and regional linkage mapping analysis was used to rapidly reduce the implicated interval, which was responsible for 20.50% of plant height variation, from 2.02 Mb to 126 kb. Interestingly, previous studies have identified plant height loci that are either near or inclusive of *qPH9*, and *qPH9* also overlaps with *qSL28-9* and *qPH9-1* (RZ422-RZ12), which are 3.49 and 7.62 Mb intervals linked to plant height (Huang et al. 1996; MacMillan et al. 2006). Meanwhile, *ph9.1* (Marri et al. 2005) and *qPH9a* (Li et al. 2003), which are also QTLs associated with mature plant height, were mapped to locations 39.66 kb and 12.44 kb away from *qPH9.* However, of all the QTLs on rice Chromosome 9 related to plant height, *qPH9* appears to be the most influential.

During the past decade, genotype datasets for many rice accessions have been released and used to identify several loci associated with important agronomic traits (Li et al. 2020; Peng et al. 2020). Several large-scale rice collections with sequence and phenotype data have provided valuable materials and knowledge for rice research and breeding projects (Li et al. 2020; Crowell et al. 2016; Zhao et al. 2018; Dong et al. 2018). Sha et al. (2021) used MBKbase-rice (www.mbkbase.org/rice) and 295 japonica cultivars from northeast China to analyze the allelic variation in *SD1*, which further enriched the allele types related to *SD1* plant height. This study found that there are multiple haplotypes in *OsPH9* in the 3 k germplasm resources and are closely related to rice plant height. It can be inferred that the mutation of *OsPH9* in CDs region 15,916,244 (T > C) is the main cause of decrease in ‘DF114’ plant height.

Histones are the basic structural proteins of chromosomes. These are alkaline in nature due to the abundance of basic amino acids Arg and Lys, thus binding tightly to the acidic DNA. Histones consist of five components with molecular masses ranging from 11 to 23 ku, which are called H1, H3, H2A, H2B, and H4 according to their molecular weights from large to small. The gene, *OsPH9*, is a H4 histone coding gene. Histone encoding genes are conservative, and among the distant species, the amino acid sequences of the four histones (H2A, H2B, H3, H4) are very similar, especially H3 and H4 histones (Mariño-Ramírez et al. 2005). Previous studies have shown that different variants of histones are closely related to the growth and development of organisms (Zhou et al. 2015; Nacev et al. 2019). The mutation of yH3 H113 in yeast caused a significant decrease in the affinity of yH3-H4 tetramer for copper ions (Attar et al. 2020). Du et al. (2020) showed *OsChz1* in rice, as a common molecular chaperone of H2A-H2B and H2A.Z-H2B, which dynamically regulates the distribution of higher eukaryotes by regulating the density of nucleosomes and the distribution of histone variants H2A.Z. Chromatin structure, which in turn regulates its developmental process. In order to further prove the function of *OsPH9* in plant height, the CRISPR/Cas9 was carried out, and the results showed that the plant height of *osph9*-mutant lines were significantly lower than the WT. These results have important significance in the field of rice plant type/plant height improvement breeding.

## Conclusion

Investigating the genetic basis of plant height is important for breeding purposes. In the present study, BSA-seq and linkage-map analysis were used to identify a QTL (i.e., *qPH9*) related with the height of a mature rice plant. Experimental verification by haplotype and sequence analysis confirmed *OsPH9* as a candidate gene of the *qPH9* region, and the function of *OsPH9* was further verified using gene-editing technology. Although further work is needed to elucidate the mechanism underlying the role of *OsPH9* in plant height, the present study provides resources for breeding programs aimed at improving rice plant height.

## Supplementary Information

**Additional file 1**.** Table S1**: Phenotypic variation of parents and F2:3 lines.** Table S2**: Number of single nucleotide polymorphisms (SNPs) and Indels detected in the samples. ** Table S3**: Statistics of variation in the 2.02 Mb interval of *qPH9* based on resequencing data. ** Table S4**: Primers used for the 13 KASP markers. ** Table S5**: Further mapping information for *qPH9* using the 13 KSAP markers. ** Table S6**: Annotated genes within the 126-Kb interval. ** Table S7**: Haplotype and plant height difference of *Os09g0433600*. ** Table S8**: Haplotype and plant height difference of chr9:15916244 T. ** Table S9**: Information on 26 germplasm resources of Hap6 haplotypes of*Os09g0433600.*

**Additional file 2**.** Figure S1**: The sequence information of *DEP1* in ‘Dongfu 114’ and ‘Longyang 11’.

**Additional file 3**.** Figure S2**: The sequence information of *Os09g0433600* in ‘Dongfu 114’ and ‘Longyang 11’.

## Data Availability

The BSA-seq data for this study can be found in the National Center for Biotechnology Information Sequence Read Archive under the accession numbers SSRR13306959, SRR13306960, SRR13306961, and SRR13306962 (https://www.ncbi.nlm.nih.gov/bioproject/PRJNA687818).

## Abbreviations

ABA: Abscisic acid
Add: Additive effects
BSA: Bulked segregant analysis
CDS: Coding sequence
Chr: Chromosome
DF114: Dongfu114
DN430: Dongnong 430
ED: Euclidian distance
GA: Gibberellic acid
KASP: Kompetitive allele-specific PCR
LY11: Longyang11
LOD: Logarithm of the odds
PVE: Phenotypic variation explained
QTL: Quantitative trait locus
RFGB: Rice functional genomics and breeding
SNP: Single nucleotide polymorphisms
