# Supplementary figures and images for: BSA-Seq and Fine Linkage Mapping for the Identification of a Novel Locus (qPH9) for Mature Plant Height in Rice (Oryza sativa)
Source: Rice (N Y). 2022 May 20;15:26. doi: 10.1186/s12284-022-00576-2 (PMC9123124; doi:10.1186/s12284-022-00576-2)

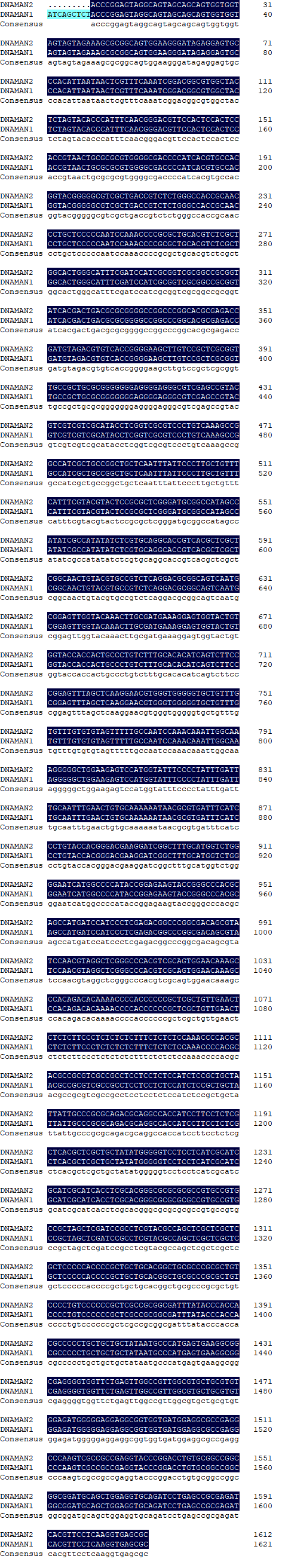

Supplement: Supplementary file 2 — Additional file 2. Figure S1: The sequence information of DEP1 in ‘Dongfu 114’ and ‘Longyang 11’. [file 12284_2022_576_MOESM2_ESM.tif]

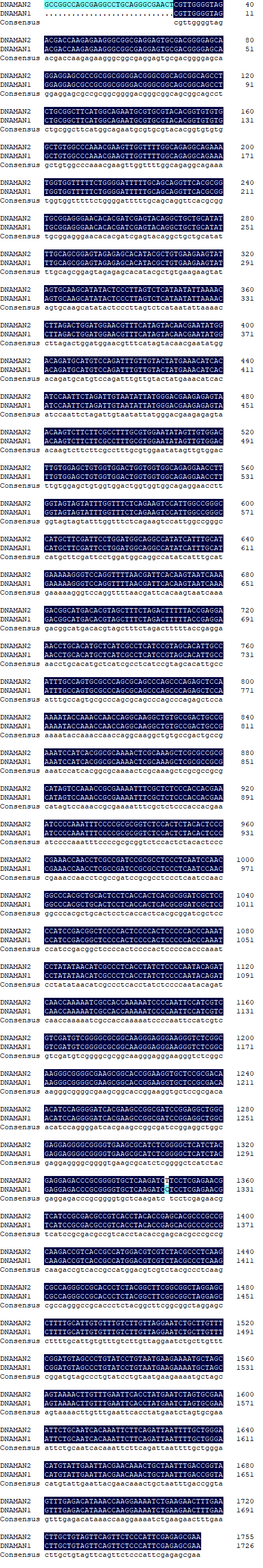

Supplement: Supplementary file 3 — Additional file 3. Figure S2: The sequence information of Os09g0433600 in ‘Dongfu 114’ and ‘Longyang 11’. [file 12284_2022_576_MOESM3_ESM.tif]
